# Tracking the Evolution of the Internet of Things Concept Across Different Application Domains

**DOI:** 10.3390/s17061379

**Published:** 2017-06-14

**Authors:** Jorge E. Ibarra-Esquer, Félix F. González-Navarro, Brenda L. Flores-Rios, Larysa Burtseva, María A. Astorga-Vargas

**Affiliations:** 1 Facultad de Ingeniería, Universidad Autónoma de Baja California. Blvd. Benito Juárez S/N, Col. Insurgentes Este. Mexicali 21270, Mexico; angelicaastorga@uabc.edu.mx; 2 Instituto de Ingeniería, Universidad Autónoma de Baja California. Calle de la Normal S/N, Col. Insurgentes Este. Mexicali 21270, Mexico; fernando.gonzalez@uabc.edu.mx (F.F.G.-N.); brenda.flores@uabc.edu.mx (B.L.F.-R.); burtseva@uabc.edu.mx (L.B.)

**Keywords:** Internet of Things, definition, things capabilities, application domains

## Abstract

Both the idea and technology for connecting sensors and actuators to a network to remotely monitor and control physical systems have been known for many years and developed accordingly. However, a little more than a decade ago the concept of the Internet of Things (IoT) was coined and used to integrate such approaches into a common framework. Technology has been constantly evolving and so has the concept of the Internet of Things, incorporating new terminology appropriate to technological advances and different application domains. This paper presents the changes that the IoT has undertaken since its conception and research on how technological advances have shaped it and fostered the arising of derived names suitable to specific domains. A two-step literature review through major publishers and indexing databases was conducted; first by searching for proposals on the Internet of Things concept and analyzing them to find similarities, differences, and technological features that allow us to create a timeline showing its development; in the second step the most mentioned names given to the IoT for specific domains, as well as closely related concepts were identified and briefly analyzed. The study confirms the claim that a consensus on the IoT definition has not yet been reached, as enabling technology keeps evolving and new application domains are being proposed. However, recent changes have been relatively moderated, and its variations on application domains are clearly differentiated, with data and data technologies playing an important role in the IoT landscape.

## 1. Introduction

The Internet of Things (IoT) has been in the spotlight for the past decade. It is regarded as one of the disruptive technologies of this century [1] and so far, has caught the attention of society, industry and academy as a way of technologically enhancing day to day activities, the creation of new business models, products and services, and as a broad source of research topics and ideas. Several alliances, institutions, enterprises and even governments have understood its importance and identified the potential benefits that can be obtained from the IoT, leading them to undertake strategic projects and initiatives aiming to develop this field and profit from it [2,3,4,5,6,7,8,9].

Though the first idea of IoT emerged no more than two decades ago, the technologies that shape and support it have been in development for many years. As its name suggests, one of the core technologies is the Internet itself, which has its origins in the ARPANET project, started in 1969 with the objectives of developing techniques and obtaining experience on interconnecting computers, improving and increasing computer research productivity through research sharing, and to permit the linking of specialized computers to the many general purpose computer centers in the U.S. Defense Department and in the private and public sectors [10]. Nowadays, the Internet is a global system of networks that interconnect computers using the standard Internet protocol suite. It has significant impact on the world as it can serve billions of users worldwide. Millions of private, public, academic, business, and government networks, of local to global scope, all contribute to the formation of the Internet [11].

Another fundamental technology for the IoT is the embedded computer system. This term was first used in 1974 and describes a computer that is physically incorporated into a larger system whose primary function is not data processing, and integral to such a system from a design, procurement and operations viewpoint [12]. These systems are implemented by using devices like microcontrollers and single board computers (SBC), and have recently gained popularity with affordable and easy to use prototyping platforms as Arduino, Raspberry Pi or Lego Mindstorms.

At the early 1990s, Mark Weiser proposed the concept of ubiquitous computing, later regarded as “pervasive”, where the main idea was for computers to be present and invisible in everything [13]. The backbone of the ubiquitous computing paradigm relies on the advances in embedded computing technologies and deploying a ubiquitous network on a scale of hundreds of computers per room. The concept might resemble that of the actual IoT, but at that time, Weiser stated that the main challenge was the design of operating systems that allow software to fully exploit the capabilities of networks, as software systems barely took any advantage of them [13].

By mid-1990s, sensor nodes started developing as several technologies like wireless communications and digital electronics presented important advances. These are tiny modules capable of sensing data, which is then processed and transmitted over a network. Large numbers of sensor nodes allow for the implementation of sensor networks and have applications in several areas [14]. Things in the IoT share some of the characteristics and purpose of sensor nodes.

However, despite all the existing work and experience related to these and other areas such as nanotechnology, big data, identification, localization and cloud technologies, there is still no consensus on the definition of the Internet of Things concept, as pointed out by several researchers and evidenced by the number of ideas and conceptions around the topic that can be found in research literature, magazines and websites of alliances, organizations and industry interested on development of the IoT. Since the term was used for the first time in 1999, its scope has widened and different definitions have been proposed, varying according to changes and creation of new technologies or the addition of old ones that have found a place within the IoT. Moreover, new terms have appeared as the IoT has extended into several domains or depending on the intended use of the technology.

Taking as a starting point this diversity of views, a systematic literature review (SLR) on the evolution of the concept of the Internet of Things was conducted. It was intended to understand the way the concept has changed and to assess the influential factors that triggered such changes. Certainly, there exist reviews, surveys and overviews that have been performed on the IoT, reflecting a considerable amount of work and analysis in order to obtain important results, but they usually try to cover a comprehensive view of the state of the art in the IoT, its enabling technologies, application areas, research opportunities and common problems. It is of our interest to focus specifically on the IoT concept and the way it is understood across different application domains, to provide a detailed report that serves as a reference for future research on the area.

The paper is structured as follows: in Section 2 the methodology for conducting the SLR is described, along with its quantitative results. The definitions and visions of the IoT found with the SLR are presented in Section 3. Next, Section 4 deals with the definitions and properties of things within the IoT. In Section 5, several related concepts and names are briefly mentioned. Results from previous sections are discussed in Section 6, and, finally, the conclusions of the study are presented in Section 7.

## 2. Methodology for the SLR

A systematic literature review (SLR) is a means of evaluating and interpreting all available research relevant to a specific research question, topic area, or phenomenon of interest [15]. Our topic of interest is the concept of the Internet of Things, the aggregated efforts to define it, the way its definition has evolved and the technologies or factors driving such evolution.

Different definitions are found in both scientific and non-scientific publications and forums and, as solutions and applications are developed in several application domains, pertaining names tend to be used. This variety of definitions and names used to refer to the same concept, reflect the existing lack of agreement on a common IoT definition. Therefore, we are conducting this SLR aiming to identify, analyze, understand, and report on the efforts that have been made to define the IoT, providing a solid background for new and current research activities in this field. This section depicts the steps undertaken in this SLR, providing the reasons for each decision and summarizing the obtained results.

### 2.1. Research Questions

Two research questions to drive the SLR were formulated as follows:

- RQ1—How has the definition of the Internet of Things evolved?
- RQ2—What are the names given to the IoT and how is it understood across different application domains?

The first question is oriented to find different definitions for the IoT and to understand how this definition has changed and what the drivers for those changes are. Realizing the IoT as a general designation for a technology, we want to identify the concepts that have been proposed in the different areas using it. So, a second question was formulated, in order to first identify such areas and then the way the IoT is referred and understood in each of them. One restriction to these questions is that we are interested in independent points of view, either coming from academic research groups, alliances or standardization agencies, avoiding any bias towards specific definitions, technologies and/or proprietary solutions from major industries or vendors.

### 2.2. Source Selections

Two main sources were selected to carry out the SLR. Both are recognized for listing and indexing publications from well renowned journals and major conferences:

- ISI Web of Science
- Scopus

Even though these sources would provide sufficient results for the SLR, four more were included as a way to provide a comprehensive and wider search. Most of their publications are indexed in the main sources, but we wanted to avoid missing important papers due to non-indexed recent creation journals or indexing delays. One example of the former is IEEE Internet of Things Journal, that was first published in 2014 and not all of its papers were found while performing searches in the main sources. The secondary sources, sorted alphabetically, are:

- ACM Digital Library
- Elsevier’s ScienceDirect
- IEEE Xplore
- SpringerLink

### 2.3. Search Strings

To answer RQ1, two search strings were structured as follows:

ST1—Documents containing the phrase “The Internet of Things” in the title. These documents were considered as highly probable to contain a definition of IoT. For this query, the following terms were avoided: “call for papers”, “editor’s note”, “guest editorial”, “special issue” and “theme issue”, because of preliminary search results did not contribute to our review.

ST2—The second search string allowed to expand the search and obtain a comprehensive set of publications about IoT. The phrase “Internet of Things” or its acronym “IoT” in the title of the documents was used. Additionally, the search was narrowed by requiring any of the words “definition”, “concept”, “evolution”, “vision”, “story” or “approach” to be present on the title, abstract or keywords.

Each of the search strings was tailored for the source search engines, and used conforming to what each of them allowed. For instance, among the selected sources, only ACM Digital Library, Elsevier’s Science Direct, Scopus and IEEE Explore support refining search by abstract and keywords.

The approach on RQ2 was different, as there was no previous indication on the number of application domains and/or possible names given to the IoT in each of them. Documents returned from the execution of ST1 and ST2 were selected, that clearly indicated a different name for the IoT. Additionally, the analysis of the literature provided a significant number of domains and names that are listed in Section 5.

### 2.4. Execution of the SLR

**Figure 1:**
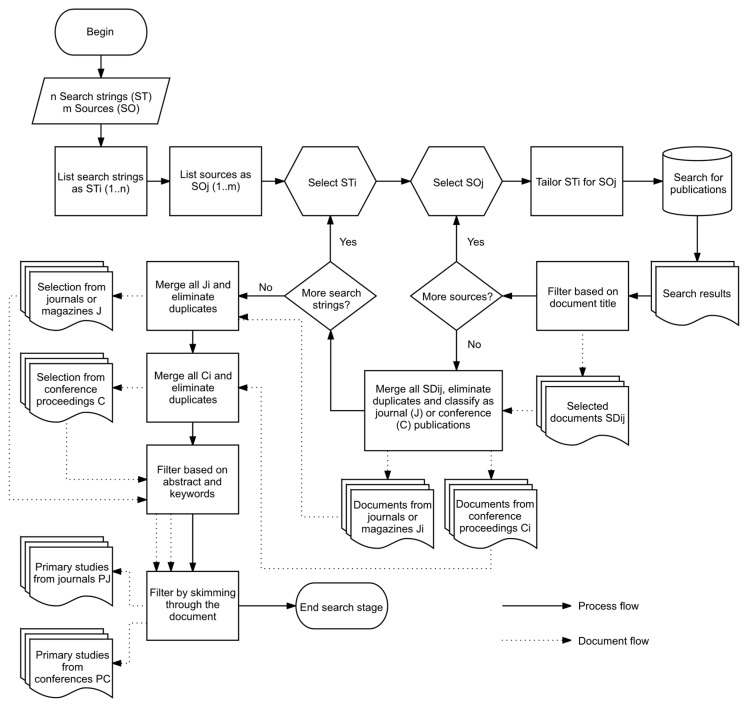
Flowchart for the search stage of the SLR.

The studies selection stage of the SLR was executed following the flowchart shown in Figure 1. The quantitative results of executing both search strings are shown in Table 1. Main sources are listed first and sorted alphabetically. Classification is made accordingly to the most common names used for types of documents in the different sources. Totals for each source and type of document are indicated in italics in the rightmost column and bottom row respectively for each search string.

A first selection of studies was based on the publication title, and classified in two disjoint sets: published in journals, magazines and reviews; and published in conference proceedings and book chapters. This classification was carried out because it is expected that the formers have passed through a more exhaustive reviewing process and results would be more suitable to the SLR’s goals. This first step was made iterating through all the sources for every search string, merging results from the different search engines and eliminating duplicates (Table 2). Results from different search strings were then merged, keeping them classified and new duplicates found were eliminated (Table 3).

The final selection of primary studies was performed in two steps. The first step involved reading the abstract and keywords, selecting those papers with a clear indication on providing a definition for the IoT or discuss its definition, and excluding those related to its architecture, reference models, related technologies, or applications in specific areas, like automotive, health or fashion. These documents were excluded from the SLR, but kept as separate sets as further stages on our planned research deal with such topics, and they could also provide an answer to RQ2.

Even though some terms in the search strings had the purpose of excluding some types of publications, we still found at this step several editorials, technology news, market reports, essays, and project descriptions. These documents were discarded, along with a few others that could not be obtained in full text.

Several documents did not provide enough information in the abstract or keywords to decide on either including or excluding them as primary studies. For these cases, a second step was performed, skimming through each document to get a clearer insight on its purpose. At the end, a set of two disjoint lists of documents {PJ, PC} was selected and its elements were used as primary studies for our SLR (Table 4), where PJ are the primary studies found in journals, magazines and reviews, and PC the ones found in conference proceedings and book chapters.

### 2.5. Studies Revision

A total of 75 studies were selected for revision, 36 in the PJ set and 39 in PC. Studies in PJ were published from 2002 to 2016, while those in PC were published from 2010 to 2016. Each study was revised to extract the following data:

- Definition of IoT, either by the authors or adherence to previously published definitions.
- Technologies that support the definition given or used.
- Application domains of the IoT, as well as the names used in each domain to refer to the IoT, if any.
- Additional literature to be included in the review.

After fully reviewing the studies, it was found that 29 of the studies in PJ and 22 in PC deal with the concept of the IoT at some extent. Several studies coincide on the lack of a common or unified definition, as the technology itself is still on a maturing stage or it is interpreted in accordance to specific needs, interests, or technical bias of a given group [16,17,18,19,20,21,22,23,24,25,26]. In addition, as expressed by Borgia, the meaning of the term continuously evolves because technology and the ideas behind it change themselves over time [18].

Some of the characteristics of the studies and journals are: The number of citations in the set of selected studies had a mean of 43.081 with a median of 3. Trimming studies with 0 citations makes a mean of 62.5 with a median of 6. In the set of discarded studies, means for each of the previous cases are 6.49 and 9.42, while medians are 1 and 2. Selected studies with at least 1 citation had a mean of 10.92 citations per year, while the discarded studies were cited 1.87 times per year. The average number of years since publication is 4.20 for all the selected studies, and 4.08 for the discarded ones. The average Journal Citations Report (JCR) impact factor (IF) for journals where selected studies were published was 2.055, most of them being in the second quartile of the JCR. The Scimago Journal Ranking (SJR) that Scopus uses to rank journals, had a mean of 0.81 for journals in the set of selected studies, with most of them being in the first quartile of this ranking. IF and SJR means for journals in the set of discarded studies resulted almost identical to means in the set of accepted studies.

The following section shows the different ideas, concepts, visions and definitions of the IoT found while analyzing the results of the primary studies from the SLR, as well as gray literature identified during the analysis. They are listed in chronological order, grouping some of the results that share some perspectives, and pointing the most relevant facts. A discussion is included afterwards.

## 3. The Internet of Things Concept

### 3.1. The Early Visions (Late 1990s to 2005)

The notion of IoT has evolved since it was conceived in the late 1990s. Joy initially proposed it as part of his six Webs taxonomy (Table 5) in a speech at the World Economic Forum in Davos (Switzerland), which was later replicated as a lecture in several technological and academic forums [27]. The sixth of such Webs is referred as device to device (D2D) and defined as an internet of sensors distributed across a mesh network, setting up urban systems for maximum efficiency [28].

Ashton first coined the phrase Internet of Things in 1999 as the title of a presentation where he linked the use of RFID in Procter & Gamble’s supply chain to the Internet. He described a vision where computers would be capable of gathering data without human help and render it into useful information, which would be possible with technologies like sensors and Radio Frequency Identification (RFID) that enable computers to observe, identify and understand the world [29].

Sarma et al. describe a world where every electronic device is interconnected and every object, electronic or not, is electronically tagged with information related to it [30]. Such tags would allow obtaining the information in a remote and contactless fashion, setting the objects as nodes in an internetworked physical world, analogous to the Internet and regarded as a new “Internet of Things.” A key element for this architecture was the Electronic Product Code (EPC) as a means to identify all physical objects and link them to the network [31]. Here, the network was understood as a seamless, ubiquitous and inexpensive system that would automatically link physical objects to the global Internet, adopting standards in cooperation with governing bodies, commercial consortiums and industry groups.

The earliest document returned by our searches where the phrase “Internet of Things” appeared as part of the title was written by Schoenberger and published in Forbes Magazine in 2002. Quoting Ashton, the IoT was deemed as a standardized way for computers to understand the real world, portraying RFID-based applications for inventory management and customer experience improvement [32].

A more comprehensive proposal was the so-called Internet-0 (I0) [33]. The potential uses and benefits of connecting everyday objects to a data network were exemplified in a series of exhibits enhanced with embedded computers and sensors. The intention with I0 was not to replace the existing Internet, but provide a compatible layer below it where connected devices depend on existing routers, gateways and name servers. Here, the original Internet idea of linking computer networks into a seamless whole was considered feasible to be extended to networks of all types of devices, a concept known as interdevice internetworking [33].

One of the earliest contributions to defining and understanding the IoT was the International telecommunication Union (ITU) Internet of Things report, published in 2005. They prospect devices and all kinds of things becoming active users of the Internet on behalf of humans, with most of the traffic flowing between them, and a number of active connections that could be measured in terms of tens or hundreds of billions. Connecting inanimate objects and things to communication networks, in addition to the deployment of higher-speed mobile networks that provide an always-on connectivity, would fulfill the vision of a truly ubiquitous network, “anytime, anywhere, by anyone and anything” [34].

The ITU portrays the IoT as a virtual world mapping the real world, where everything in our physical environment has its own identity in virtual cyberspace, thus enabling communication and interaction between people and things, and between things. This vision is based on the application of key technological enablers that would account for an expanded Internet, able to detect and monitor changes in the physical status of connected things in real-time [34].

### 3.2. Establishment Phase (2009 to 2011)

Building on the previous ideas, the concept starts changing from what can get connected to the network, to what can be done with the things that are connected to the network. Haller et al. state that the role of the Internet of Things is to bridge the gap between the physical world and its representation in information systems [35]. In accordance, they define the IoT as “A world where physical objects are seamlessly integrated into the information network, and where the physical objects can become active participants in business processes. Services are available to interact with these smart objects over the Internet, query their state and any information associated with them, taking into account security and privacy issues”.

Based on the belief of continuous and steady advances in microelectronics, communications and information technology, Mattern and Flörkemeier see the Internet extending into the real world embracing everyday objects [36]. Physical items connect to the virtual world where they are controlled remotely and can act as physical access points to Internet services.

The gap between the virtual and physical world is bridged by several technical developments taken together. Such developments provide the objects with capabilities like communication, cooperation, addressability, identification, sensing, actuation, embedded information processing, localization, and novel user interfaces, which contribute to an evolution of the IoT paradigm that started with the remote identification of objects to a system where smart objects actually communicate with users, Internet services and even among each other. More than defining the IoT, they describe the features of the objects that get connected in the IoT, a characteristic commonly found in the reviewed papers, which is discussed in Section 4. Two definitions that are commonly adopted by researchers or used as a starting point for their own definitions are the results of European Commission promoted initiatives. The first of these concepts was determined within the CASAGRAS project as [37]:

“A global network infrastructure, linking physical and virtual objects through the exploitation of data capture and communication capabilities. This infrastructure includes existing and evolving Internet and network developments. It will offer specific object-identification, sensor and connection capability as the basis for the development of independent cooperative services and applications. These will be characterized by a high degree of autonomous data capture, event transfer, network connectivity and interoperability.”

Then, the European Commission, through the Cluster of European Research Projects on the Internet of Things (CERP-IoT), defines the IoT as “a dynamic global network infrastructure with self-configuring capabilities based on standard and interoperable communication protocols where physical and virtual things have identities, physical attributes, and virtual personalities and use intelligent interfaces, and are seamlessly integrated into the information network” [38].

**Figure 2:**
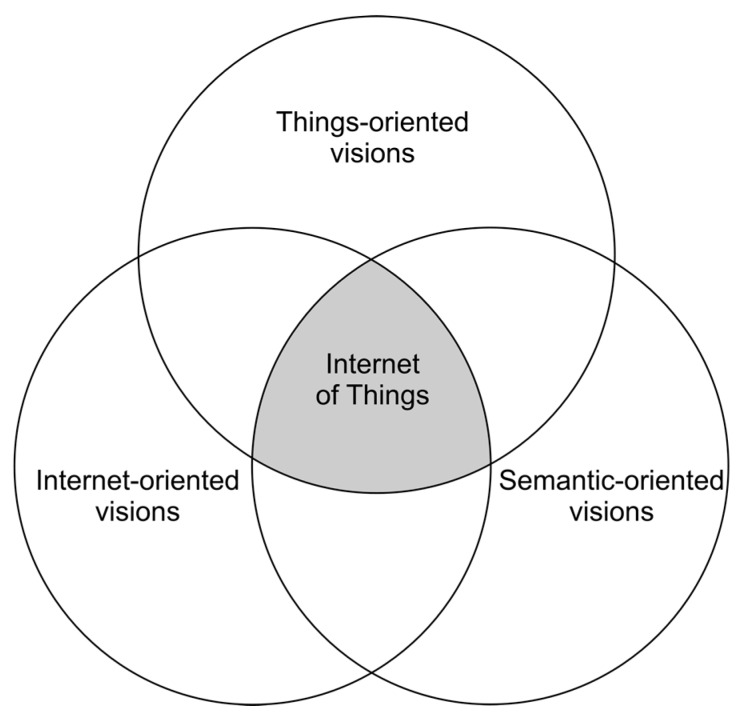
Three main visions of the IoT. Adapted from [16].

Atzori et al. discussed several interpretations and definitions of the IoT [16]. They state the basic idea of this concept is the pervasive presence around us of a variety of things or objects—such as RFID tags, sensors, actuators, mobile phones, etc., which, through unique addressing schemes, are able to interact with each other and cooperate with their neighbors to reach common goals [39]. In their analysis, the IoT paradigm is depicted as the result of the convergence of three main visions: Things-oriented, Internet-oriented, and Semantic-oriented—see Figure 2 [16].

Under this paradigm, three fundamental building blocks of the IoT concept can be inferred: the way objects or things get connected to the network, the capabilities of interaction and communications provided by the latter, and the use and interpretation of the information provided by things. As the authors in [16] suggest, the IoT has the potential to add a new dimension to the process of interaction between people and machines, by enabling communications with and among smart objects, thus leading to the vision of ‘‘anytime, anywhere, any media, anything” communications.

Huang and Li analyzed the semantic meaning of the phrase Internet of Things to understand the properties of IoT [40]. They see it as a network for globally sharing information about things, which they refer to as products. After reflecting on both the literal and intrinsic meaning, they propose a semantic meaning for IoT as a global system for sharing product information among interconnected products.

A similar approach is offered by Zhang et al., defining the IoT as a network that uses sensing and localization devices attached to things, or articles, connected to the Internet for information exchange and communication, to achieve intelligent recognition, location, tracking, monitoring and management functions [41]. They understand it as an extension and expansion of the existing Internet, which remains as the core and foundation of IoT, with articles that make use of clients for Internet services.

According to Ma, the IoT can enable the interconnection and integration of the physical world and the cyberspace; representing the trend of future networking, while leading the third wave of the IT industry revolution [42]. Comparing IoT with Wireless Sensor Networks (WSNs), Internet, ubiquitous networks and further analysis, the author defines IoT as a network that interconnects ordinary physical objects with identifiable addresses, providing intelligent services. The IoT is based on traditional information carriers like the Internet or telecommunication networks. Furthermore, the instrumentation of ordinary objects, their interconnection through autonomic terminals, and providing intelligent pervasive services are listed as important characteristics of the IoT.

There are visions where the IoT is perceived as an extension to the existing Internet. This is supported by the fact that, in the IoT, not only subjects but also objects will be connected and enabled to exchange and share information. In addition, the development of the IoT is considered as one of the manifestations of the concept of ubiquitous computing [43].

Coetzee and Eksteen see the IoT as part of the Future Internet [44]. They describe a vision where objects become part of the Internet: where every object is uniquely identified, and accessible to the network, its position and status known, where services and intelligence are added to this expanded Internet, fusing the digital and physical world, ultimately impacting on our professional, personal and social environments. The driver for the IoT is an expansion of the Internet through the inclusion of physical objects combined with an ability to provide smarter services to the environment as more data becomes available.

### 3.3. A shift Towards Data and Services (Starting in 2012)

In most recent definitions and visions, the role of things as data producers and the network as enabler of new services are highlighted. In their work, Miorandi et al. see the IoT as an extension of the existing Internet and the Web into the physical realm [45]. As they state, the IoT uses the Internet as a global platform for letting machines and smart objects communicate, dialogue, compute, and coordinate. These objects act as providers and/or consumers of data related to the physical world, giving the IoT a focus on data and information rather than on point-to-point communications. They define the IoT from a system-level perspective, as a highly dynamic and radically distributed networked system, composed of a very large number of smart objects producing and consuming information.

With a different perspective, Mayordomo et al. envisage the IoT as a sort of evolution of the Internet to reach the physical everyday objects. They define it as a new dynamic network of networks, where every daily object can communicate to each other [46].

Lee et al. see the Internet evolving from a network of interconnected computers to a network of interconnected objects, bolstered by the combination of several technologies including embedded microcontrollers, sensors, actuators, network interfaces, and the greater Internet [47]. They recognize three principal viewpoints from where different interpretations of the IoT emerge: Internet or network oriented; Objects or things oriented; and complex distributed systems. In the opinion of Lee et al., these diverging views have brought many attempts to define IoT that it results advisable to move forward with a common understanding in a global perspective.

Gubbi et al. discuss that although the definition of “Things” has changed as technology evolved, making it more inclusive in order to cover a wide range of applications, the main goal of making a computer sense information without the aid of human intervention has remained the same [48]. This represents an evolution of the current Internet into a network of interconnected objects, able to harvest information from the environment, interact with the physical world and use existing Internet services for information transfer, analytics, applications and communications.

Building on previous works in the field of ubiquitous computing, they provide a user-centric definition for the IoT in the context of smart environments, as the “interconnection of sensing and actuating devices providing the ability to share information across platforms through a unified framework, developing a common operating picture for enabling innovative applications” [48]. Though the definition might seem too general, the authors claim its purpose is to allow development and deployment of long-lasting applications using the available state of the art protocols at any given point in time, enabled by seamless ubiquitous sensing, data analytics and information representation with cloud computing at the center of a conceptual IoT framework.

In June 2012, the ITU approved recommendation ITU-T Y.2060, which provides an overview of the IoT and has the objective of highlighting it for future standardization. As part of the recommendation, they define the IoT as “a global infrastructure for the Information Society, enabling advanced services by interconnecting (physical and virtual) things based on, existing and evolving, interoperable information and communication technologies”, understanding it as a far-reaching vision with technological and societal implications [2]. This definition has since been adopted by several researchers, as it is supported by a comprehensive reference model updated from their 2005 report [34].

Another aspect that gets considered in IoT conceptualizations is that of intelligence. Mzahm et al. introduce it through the concept of Agents of Things, understanding the IoT as the enabler for objects or things to connect and communicate with other objects in the world via the Internet [49]. However, they find some deficiencies as things lack the ability to reason on their environments and make intelligent decisions and actions to achieve their objectives. Accordingly, they infer the system as a whole may be represented as being intelligent but not individual things. This interpretation is shared by Chen et al., who describe the IoT as an intelligent network which connects all things to the Internet for exchanging information and communicating through the information sensing devices in accordance with agreed protocols [19].

Such protocols need not to be reinvented. As suggested by Aggarwal et al., if the objects are uniquely addressable and connected to the Internet, then the information about them can flow through the same protocol that connects our computers to the Internet [50].

Some visions of the IoT highlight the business impact of the IoT as a provider of new services. Alam et al. see the IoT evolving from the field of Machine to Machine (M2M) communication, adding value to services and applications for businesses relying on the M2M value chain, taking advantage of the network infrastructure and computing capabilities to improve operational and business processes and enhance customer experience [51]. As the authors describe it, “the IoT to be seen tomorrow is a concept that moves beyond the basic connectivity and technological innovations and merges the gap to the envisioned use cases in order to bring the needed functionality and business values. This includes a focus on platforms and how solutions are delivered with horizontal platforms that are able to support a multitude of vertical solutions”.

Chaouchi et al. describe the IoT as a provider of new services to networked and connected objects, which in turn will provide services to persons. They imply the Internet model must be adapted to support the connectivity and traffic transport of the new services based upon the connected objects [52].

For Skaržauskienė and Kalinauskas, the main idea of the IoT is circling around a connected network or networks in which things and other sensor-based objects communicate with each other. The ability to integrate small, energetically efficient and cheap sensor based objects into clouds of networks would cause the new business and service models and would expand beyond the human-machine interaction approach which is most frequently used in Internet-based environments [53].

### 3.4. Things as a Key Element

Even though it has been over a decade of evolution for the IoT, in recent publications the IoT is still considered to be in its early stages. As Borgia points out, IoT refers to an emerging paradigm consisting of a continuum of uniquely addressable things communicating one another to form a worldwide dynamic network [18]. Chen states that in the future, digital sensing, communication, and processing capabilities will be ubiquitously embedded into everyday objects, turning them into the IoT. In this new paradigm, smart devices will collect data, relay the information or context to each another, and process the information collaboratively using cloud computing and similar technologies. Finally, either humans will be prompted to take action, or the machines themselves will act automatically [54]. As a long-term vision, the trend of IoT is the fusion of sensing and Internet, where all the networked things are flexible, smart, and autonomous enough to provide required services [55].

In 2015, the results of a study aimed to define the IoT were published at the IEEE IoT Initiative Web portal. Authors proposed two definitions depending on size and complexity of systems. For low complexity systems, they define the IoT as a network that connects uniquely identifiable things to the Internet, where the things have sensing/actuation and potential programmability capabilities and, through the exploitation of unique identification and sensing, information about the thing can be collected and its state can be changed from anywhere, anytime, by anything. For global distributed systems, where a large number of things can be interconnected to deliver a complex service and support an execution of a complex process, they propose the following definition of IoT [56]:

“Internet of Things envisions a self-configuring, adaptive, complex network that interconnects ’things’ to the Internet using standard communication protocols. The interconnected things have physical or virtual representation in the digital world, sensing/actuation capability, a programmability feature and are uniquely identifiable. The representation contains information including the thing’s identity, status, location or any other business, social or privately relevant information. The things offer services, with or without human intervention, through the exploitation of unique identification, data capture and communication, and actuation capability. The service is exploited through the use of intelligent interfaces and is made available anywhere, anytime, and for anything taking security into consideration.”

This definition encompasses many of the previously found definitions, but we see it more as a comprehensive description of the IoT than a definition. However, viewing the IoT according to the complexity and size of the systems could actually lead to a better understanding and ease implementation of solutions at different scales.

Some recent definitions revolve around four basic actions: identifying, sensing, networking and processing. The use of technology required for those actions is evolving in terms of the number and kinds of devices as well as the interconnection of these devices across the Internet. The IoT proposes to attach this technology to everyday devices and making them online, even if they were not initially designed with this capability in mind [22]. Hurlburt describes a model for the IoT involving sensing, thinking, and acting, which usually occur iteratively in that order. He compares sensing to the human five primary senses, thinking to the way the human brain processes information, and acting to interaction, which represents a difference to the concept of autonomy proposed by others [24]. Despite proposing a definition mostly around the interconnection of objects, Pintus et al. support the need for interaction between people and things, which they present as a humanized view of the IoT [57].

Finally, a pair of definitions were found that, as others before, take a data approach, but stress on what is done with data and how it is transformed into knowledge via data mining techniques. Dorsemaine et al. define the IoT as a group of infrastructures interconnecting connected objects and allowing their management, data mining and the access to the data they generate [23]. They identify a connected object as a device equipped with sensors and/or actuators carrying out a specific function and being able to communicate with other equipment. Authors back their definition with a four-layer architecture.

On their part, Qin et al. focus their study of the Internet of Things from a data perspective, considering that data is processed differently in the Internet of Things and traditional Internet [11]. In the Internet of Computers, both main data producers and consumers are human beings. However, in the Internet of Things, the main actors become things, which means things are the majority of data producers and consumers. Therefore, computers will be able to learn and gain information and knowledge to solve real world problems directly with the data fed from things. As a goal, computers enabled by the Internet of Things technologies will be able to sense and react to the real world for humans.

## 4. Things in the IoT

An imperative component of the IoT are the objects that get connected to it, or simply things. Characterizing them helps understanding the capabilities and possibilities of the IoT and that's why many researchers have made an effort in describing and defining things as a means to express their conception of the IoT. As pointed by Coetzee and Eksteen, the definition of things in the IoT vision is very wide and includes a variety of physical elements, like personal objects we carry around such as smart phones, tablets and digital cameras. It also includes elements in our environments as well as things fitted with tags which become connected via a gateway device [44]. In this section, several definitions, descriptions and properties of things, as found in the analysis phase of the SLR, are presented.

### 4.1. Names Given to Things

Within the context of the Internet of Things, a thing is defined as a real/physical or digital/virtual entity that exists and moves in time and space and that can be identified [43]. This definition reminds of that of the spime, presented by Bruce Sterling in his book “Shaping Things”. He refers to spimes as manufactured objects whose informational support is so overwhelmingly extensive and rich that they are regarded as material instantiations of an immaterial system. These are digital objects that can be tracked through space and time and contain the data history related to the specific object they represent. Therefore, a key element to the spime is identity, i.e. a spime must be a thing with a name [58].

A more commonly used name is the smart object. Kopetz considers smart objects the building block of the IoT and describes them as everyday physical things that are enhanced by a small electronic device to provide local intelligence and connectivity to the cyberspace established by the Internet [59]. Aggarwal et al. see smart objects as examples of the spime, describing them as tiny computers which have sensors or actuators, and a communication device [50]. Smart objects are also defined by their characteristics, as objects that [45,60]:

- Have a physical embodiment and a set of associated physical features.
- Possess a unique identifier.
- Are associated to at least one name and one address.
- Can sense and store measurements made by sensor transducers associated with them.
- Have a minimal set of communication functionalities that allow them to make their identification, sensor measurements, and other attributes available to external entities such as other smart objects or systems.
- May possess means to trigger actions having an effect on the physical reality.
- Possess some basic computing capabilities which can be used to make decisions about themselves and their interactions with external entities.

Usually, things are referred to just as objects. An object in the IoT is regarded to as any machine, device, application, computer, virtual or physical object involved in a communication that could connect to the Internet, and could have the ability to create, request, consume, forward or have access to digital information [61]. There are similar concepts often mentioned in literature like smart parts, smart items or intelligent products.

### 4.2. Properties of Things

Several properties of things are found in the literature, often with different names but implying the same meaning. In this section, the properties that better describe things in the IoT are listed.

#### 4.2.1. Identification

This property dates to the initial visions of the IoT where each object was meant to include an RFID tag allowing it to be uniquely identified. In the ITU’s 2005 report, they mention RFID provides the means for location-specific item identification that is fundamental to thing-to-thing communication, implying that tagging virtually every object on earth with an RFID tag would become feasible, and deeming RFID as a key enabler of the IoT [34]. With a more general perspective, Sánchez López et al. consider automatic identification technologies as fundamental to the realization of the IoT because they enable things to be linked with their virtual identity on the Internet [60]. Miorandi et al. mention three pillars on which the IoT is built, the first of them being the ability of smart objects to be identifiable [45].

Additionally, Borgia suggests this identification should be based on assigned numbers, names, or addresses [18]. Miorandi et al. say things are associated to at least one name and one address, where the name is a human-readable description of the object and can be used for reasoning purposes, while the address is a machine-readable string that can be used to communicate to the object [45]. Moreover, the vision of the IoT, where billions of smart objects can communicate via the Internet, requires a well-thought-out naming architecture to be able to identify a smart object and to establish an access path to the object [59].

#### 4.2.2. Location and Tracking

As huge amounts of objects get connected to the IoT, and provided they can be uniquely identified, individual objects will be tracked, its condition and location communicated in real time to a higher-level service [44]. Chaouchi et al. present a classification of objects, adapted from the CASAGRAS project, where they consider aspects of movability, with objects that are inherently mobile and need to be tracked, while others maintain a fixed position and this property is not needed [52]. The way things get connected to the IoT, either wired or wireless, provides a lead to where they might stand in this classification and their need to be tracked. However, as implied by Speed, tracking a thing not only refers to being aware of its physical location, but also to its individual history, since manufacturing to the end of its lifetime [62]. This way, a single thing can provide useful information that wouldn’t be available otherwise.

#### 4.2.3. Sensing

This property refers to the ability of things to collect data from the environment. The ITU names things equipped with sensors as “feeling things” and consider sensors complement human senses [34]. The use of sensors as a key element of the IoT was introduced in the first visions proposed in the late 1990s [28,29], though the earliest implementations of the IoT focused mainly on identifying, locating and tracking objects. With sensors, things can become aware of their characteristics, context and situation [47]. This is, a thing not only provides information about its environment, but also about its status.

#### 4.2.4. Actuation

By means of actuators, things can influence their environment [44]. This actuation can be based on sensed data and controlled remotely via the Internet [18,63] and is fundamental for automation of processes.

#### 4.2.5. Processing

The property of processing data and executing commands is frequently mentioned as intelligence. The ITU describes “thinking things” in reference to materials and things that get labeled as smart [34]. Sánchez López et al. describe it as embedded processing for local intelligence and autonomy [60]. In [63], Xue et al. give the denomination of robots to things that perform intelligent computing and execute agents. On their part, Razzaque et al. imply that as the processing capabilities of things improve, they can become not only providers of data but also of services [64].

Additional properties are mentioned in literature, though we don’t consider them to be critical to objects in the IoT. However, an interesting feature is proposed by Jazayeri et al., for IoT devices to easily plug and play, each IoT device needs to be self-describable and self-contained in order to communicate with other objects or services, so they can describe and advertise themselves and their capabilities [65]. They also imply a need for interoperability, as communication protocols and data encoding for current IoT devices are usually proprietary and different from each other.

### 4.3. Things Abstracted

By taking the aforementioned properties as capabilities of things, they can be abstracted as devices in overlapping sets that possess one or more of such capabilities. In the context of the IoT, a thing is considered a physical object with attached, embedded or built-in electronic devices with Internet connecting capabilities. Considering as a requirement being connected to the Internet, things can be categorized as follows, where at least one of the first four capabilities is required to be part of the IoT:

- Identification capability (IC): Things that can be uniquely and unmistakably identified, either by an electronic tag, hard-coded serial number or printed label that is read by another object.
- Localization capability (LC): Things that know their precise physical location in the world by their own means, e.g. using embedded geolocation devices, and can communicate it to other things and services.
- Sensing capability (SC): Things equipped with sensors to obtain data from their actual state or the environment. They may or not include temporary storage capabilities or make use of cloud-based storage services.
- Actuation capability (AC): Things equipped with actuating devices that can be remotely controlled to modify the environment.
- Processing capability (PC): Things that can process information obtained by themselves or received via the Internet. Connected devices with processing capabilities but none of the previous, are considered as part of the Internet of Computers (IoC), but not the IoT.

**Figure 3:**
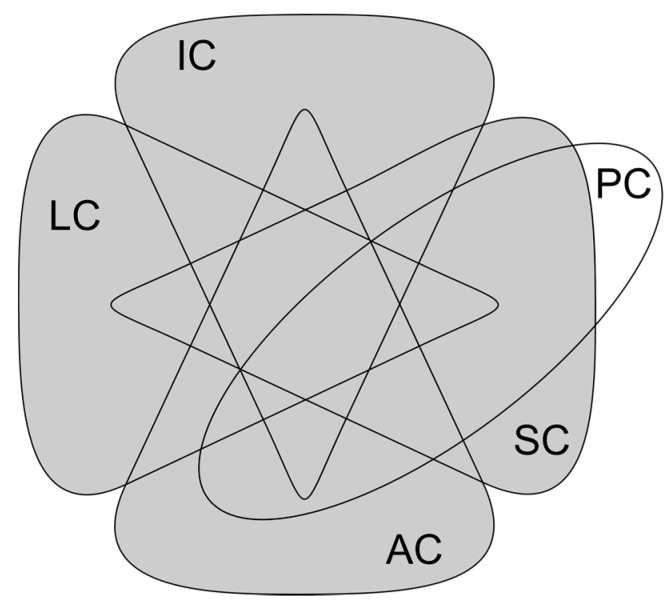
Capabilities of Internet connected devices as sets. The shaded area represents the set of Things in the IoT.

**Figure 4:**
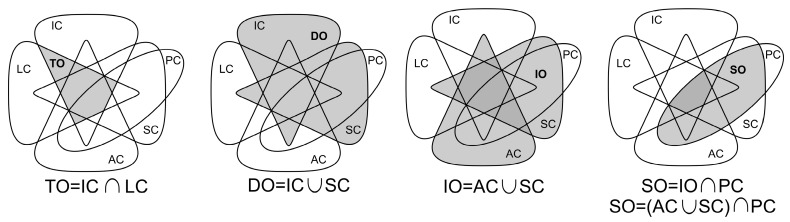
Subsets of the IoT.

Figure 3 presents an Euler diagram with the relationships between the capabilities of Internet connected devices. Devices with any of the capabilities in the shaded area of the diagram are considered part of the IoT. The diagram shows that any combination of the first four capabilities is possible, but having processing capabilities in things that can only be identified or located is not seen as an added value, as these types of things only share information about their characteristics or physical location, without creating or transforming data from their environment or received from the Internet. From this, four subsets that result of interest in the IoT are identified and shown in Figure 4.

The subsets are described as follows:

- Trackable Objects (TO): Mobile things that can be uniquely identified and are aware of their physical location.
- Data Objects (DO): Things producing data either from sensors or their current properties or state.
- Interactive Objects (IO): Things that allow an interaction with the environment where they are immersed, either by measuring environmental variables, modifying the environment or both. In the figure, the darker area represents the latter type of things.
- Smart Objects (SO): Interactive things that can apply some degree of processing to data obtained or received and act accordingly. Objects in the darker area have the five capabilities and are the most comprehensive devices in the IoT.

## 5. Other Names Given to the IoT

As the IoT extends into several application contexts, so does the way it is named and understood. This section presents the names given to the IoT in such contexts, as found in the reviewed literature, accompanied by a brief description. Some of these names represent complete new concepts on their own, while others are mere specializations of the IoT.

The Web of Things (WoT) is a concept described in [66] as making things web-present by embedding web-servers in them or by hosting their web-presence within a web server. The name was first used as part of Sun Microsystem’s project JXTA [67], defining a set of protocols for building applications and deploying them on a virtual network. More recently, Guinard and Trifa proposed an architecture for making devices an integral part of the Web by using HTTP as an application layer [68]. In this context, the term Web of Objects is also used [52] as well as Physical Web [69]. It is important to note that all of the Web-based visions consider naming services of things as an important feature.

Extending from the IoT and the WoT, the notion of Wisdom Web of Things (W2T) represents a holistic intelligence methodology for realizing the harmonious symbiosis of humans, computers, and things in the hyper world. This concept relies on different abstractions of intelligence and the creation of knowledge from data. The word “wisdom” implies that each thing in the WoT can be aware of both itself and others to provide the right service for the right object at a right time and context [70].

A frequently used name is the Future Internet of Things (FIoT), based on the gradual development and incorporation of several innovative techniques into the IoT. Among these techniques, how to extract data from sensing and transfer it into knowledge is usually found [21]. The term Future Internet is often used to make reference to the future conditions and applications that will be available through the Internet.

In [49], The Agents of Things (AoT) concept is proposed to mitigate the effect of the loT deficiencies and limitations in terms of intelligence. The core idea of AoT is that everything in this concept should have an internal reasoning and intelligence capability, enabling the things to interact directly with other things in the same or different system types.

Building on the idea of adding intelligence to the IoT, the concept of Cognitive Internet of Things (CIoT) is proposed. A CIoT is an IoT with cognitive capability which is integrated to promote performance and achieve intelligence. Given that there is a business process flow corresponding to a given application, CIoT comprehends current business types and network conditions, analyzes the perceived information based on the prior knowledge, makes intelligent decisions, and performs adaptive and control actions, aiming to maximize network performance and meet the application requirements [71].

A different approach is that of the Social Internet of Things (SIoT), which is based on a sort of social relationship among objects, analogous to what happens for human beings. The SIoT represents an innovative paradigm of interaction among objects, where the basic idea is the definition of a “social network of intelligent objects” [72].

The human perspective of the social aspect of the IoT is represented in the Internet of People (IoP), envisaged as people becoming part of ubiquitous intelligent networks having the potential to seamlessly connect, interact and exchange information about themselves and their social context and environment [73]. Integrating the notions of the IoT, IoP and SIoT allows to present the idea of a Humanized Internet of Things (HIoT), which enables interactions between communicating entities: smart things and people, the physical world and the digital one [57].

The interaction with the environment is also important for any type of system, and that is represented in the Green IoT. It is defined as the energy efficient procedures, either in hardware or software, adopted by IoT to facilitate reducing the greenhouse effect of existing applications and services or to reduce the impact of greenhouse effect of IoT itself [74].

A concept that is used along the IoT is Cyber-Physical Systems (CPS). CPS can be considered a confluence of embedded systems, real-time systems, distributed sensor systems and controls [75]. They are integrations of computation with physical processes by means of embedded computers and networks that monitor and control the physical processes, usually with feedback loops where physical processes affect computations and vice versa [76].

CPS are physical systems designed with the electronic devices for communications, sensing, and controlling as a part of them and hence invisible to the user, providing a sense of immediacy; in the IoT, these devices may be embedded into existing physical systems to get them connected. Hence, some authors identify the IoT as a subset of CPS [18,77].

One of the most popular designations is the Industrial Internet of Things (IIoT) or just Industrial Internet. This is a form of IoT where the devices, or things, are objects in manufacturing plants, dispatch centers, process control industries, etc. [78]. This name, proposed by General Electric, is mostly used in North America, while European initiatives usually rely on the German designation of Industry 4.0, referring to the fourth industrial revolution and often understood as the application of the generic concept of CPS to industrial production systems (cyberphysical production systems) [79].

Several other fields are adopting the IoT and specific interpretations are being proposed. The idea in each case is to take advantage of the sensing, actuating, communications, data processing, identification, and interaction capabilities of objects pertaining to each application domain. Some of the most commonly found in the literature are the Internet of Vehicles [80], Health Internet of Things [81,82], Internet of Personal Health (IoPH) [83], Internet of m-health Things (m-IoT) [84], Internet of Agriculture [85], and Agriculture Internet of Things [86,87].

## 6. Discussion

Two clear trends were identified in the literature: some authors describe the IoT as an extension to the existing Internet, while others present it as an evolution of the Internet. We find it is important to make a distinction, as the first idea represents that new technology, devices, applications and services are being added and made available through the Internet, while the second implies a progressive change that would end in a replacement of the existing technology. These diverging views clearly represent some of the reasons why this SLR was conducted, and exemplify the lack of consensus on defining the IoT expressed by several authors [16,17,18,19,20,21,22,23,24,25,26].

**Figure 5:**
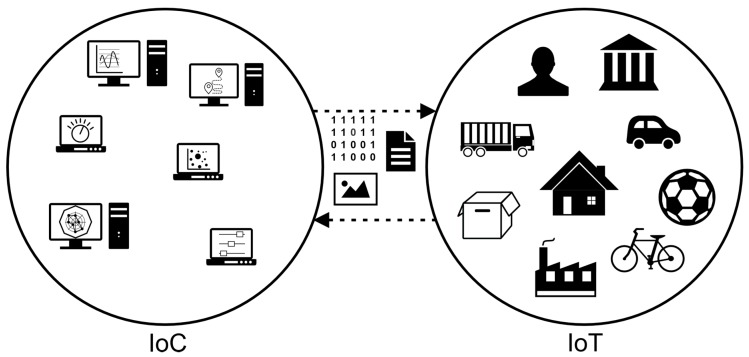
Interaction between IoC and IoT devices.

What can be inferred from the literature is that the Internet of Computers (IoC) and the Internet of Things (IoT) are complementary entities in an ecosystem of Internet-connected devices, providing data and services for each other. Therefore, even though in terms of devices the IoC and IoT can be seen as two disjoint sets in a universe of Internet reachable devices, in terms of data and software, devices in the IoC function as means for accessing those in the IoT for configuration, modification, and data storage, processing and visualization. Figure 5 displays examples of devices and applications that can be found in each of these sets, sharing different types of data, like binary strings containing commands or raw data, documents in application-specific formats, or images. By means of their networking capabilities, elements in both sets can communicate to each other and take advantage of their specific characteristics and features, and the services they provide.

Definitions of the IoT tend to be centered on distinct aspects. The initial notions of Ashton and Joy made clear references to the capabilities of things to sense data from the environment. However, for most of the first decade the focus of the definitions was on networking issues and RFID-based services. Later, desired characteristics of communications were integrated into the concept, and the view on services shifted towards business processes, new business models and customer experience, describing services as independent, cooperative, intelligent or advanced. The work of Atzori et al. [16] is the first to explicitly define the IoT in terms of the networks requirements for connecting things, their capabilities for communications and interaction, and the use and interpretation of data.

**Figure 6:**
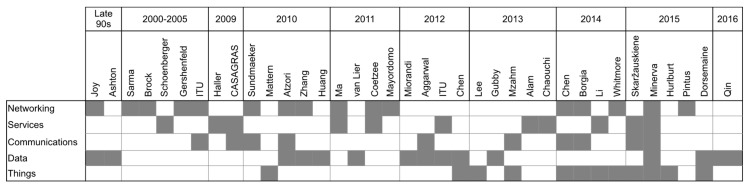
Orientation of the definitions and visions of the IoT.

More recent definitions, while still mentioning network and communications characteristics, are more oriented to describe the capabilities and properties of things and the importance of harvesting and processing data that can be turned into knowledge for the improvement of business processes. Figure 6 shows where definitions and visions of the IoT were centered in the selected studies (Only the last name of the first author is shown).

**Figure 7:**
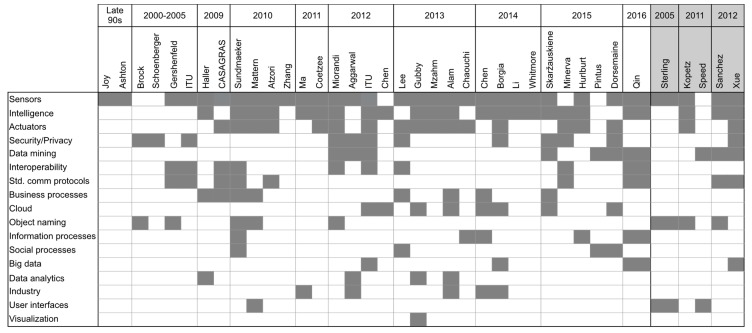
Concepts and notions used by authors in presenting their visions of IoT.

In Figure 7, the most relevant concepts and notions that were used by authors in explaining their visions of IoT are listed. Terms like identification, location, tracking, and specific technologies like RFID were taken off the list as we consider they don’t provide additional information to what has been said before. Also, most of the authors make use of specific applications or whole application fields as to exemplify and describe the potential impact of the IoT. The last three columns, shadowed in light gray, correspond to publications where the authors describe the characteristics and properties of things instead of a definition or vision of the IoT. Some of the publications shown in Figure 6 do not appear in Figure 7, as the selected concepts are not used by their authors.

The three most recurring concepts are sensors, intelligence, and actuators. While sensing capabilities have been considered since the first interpretations of the IoT, it is not the case with intelligence and actuators. Intelligence in the IoT is used in several contexts, but its common understanding is the ability to process data, which in its most common use refers to data processing. Concepts like security, privacy, interoperability, and the need for standard communication protocols are often used in presenting the visions, but they are seldom included in the final definitions.

The execution of data mining processes is seen as essential for the IoT in recent definitions, as well as the use of cloud technologies for providing services and storing data. This can accelerate knowledge acquisition via information processes and, with the aid of data analytics and visualization techniques improve the outcomes of both business and social processes mediated by things in the IoT, which also will have important benefits for industry, an actor portrayed as one of the main participants in the future development of IoT.

As explained in most of the descriptions of the IoT, including those specific to different application domains, a huge number of things will be or have already been deployed, generating data that should be treated with technologies and algorithms for big data, which is also mentioned by some authors. For all connected things, two important factors are for end users to be able to easily find and use them. As we are used to find a website by its name, finding a thing by its name may appear as an obvious choice, and so it is proposed by several authors. On the other side, not many of them seem to pay special attention to the final user means of interaction with things, but those who are concerned with describing things and their properties emphasize the importance of designing proper user interfaces.

## 7. Conclusions

The Internet of Things is the confluence of several technologies that allow providing Internet-based services and applications supported by electronic devices attached to physical things for acquiring data and controlling processes. As a general understanding, this phrase might well describe the IoT, but the variety of definitions and visions found in literature prove defining a complex technology or range of technologies is also a complex process.

However, though these definitions might appear as diverging, they are usually presented around five elements, occasionally including more than one of them: networking, services, communications, data and things. Earlier definitions were more commonly centered on networking aspects, while the most recent tend to be more comprehensive, as expected when a technology is in a maturing stage and its scope begins widening and more capabilities and possibilities are discovered.

Conducting a SLR on the concept of the IoT allowed us to obtain a clearer insight on this technology and what can be achieved through it. Observing the way these definitions have evolved and how different concepts, technologies and ideas have been incorporated as the IoT develops suggests that a correct description and characterization of the things at the end-points of the IoT should be one of the first goals towards a final definition. Latest efforts are paying more attention to things and what things can do as part of new services, applications and business models inspired by the IoT.

Several visions and definitions of the Internet of Things were found and analyzed. They can be categorized in different ways, as to how the technology is understood, which part of the IoT spectrum is the definition biased to, or the technologies that are mentioned as fundamental part of specific visions. These definitions are extended and complemented with specific terms of the different application domains of the IoT, often with designations that researchers adapt to each domain.

Just as important is how data gets immersed in the whole concept. Enormous amounts of different data will be gathered from a myriad of sources, and a lot more can be inferred from what is directly obtained by sensors. One of the main goals of the IoT should be the creation of new data and obtaining data that couldn’t be obtained otherwise. So, advanced and novel data mining, big data, and data analytics techniques and algorithms will be required to treat this data and shouldn’t be excluded from a definition of the IoT.

## Figures and Tables

**Table 1:** Results of executing ST1 and ST2 in each of the selected sources.

**Search String 1**				
**Base String**	“The Internet of Things”			
**NOT**	“call for papers” “editor’s note” “guest editorial” “special issue” “theme issue”			
**Results**	Journal/Magazine	Conferences/Book chapters	Reviews	Total
ISI	236	0	16	252
Scopus	470	909	38	1417
ACM	18	98	0	116
Elsevier	85	0	0	85
IEEE	111	571	0	682
Springer	46	192	0	238
	*966*	*1770*	*54*	**2790**
**Search String 2**				
**Base String**	“Internet of Things” OR “IoT”			
**AND** (Title+Abstract+keywords)	“definition”	“concept”	“evolution”	“vision”
	“story”	“approach”		
**Results**	Journal/Magazine	Conferences/Book chapters	Reviews	Total
ISI	42	0	2	44
Scopus	464	973	25	1462
ACM*	24	138	0	162
Elsevier	16	0	0	16
IEEE*	115	949	0	1064
Springer	92	310	0	402
	*753*	*2370*	*27*	**3150**

**Table 2:** First selection stage.

**Selection by Document Title (ST1)**					
	**Journal/Magazine**	**Conferences/Book Chapters**		**Reviews**	**Total**
ISI	40	0		6	46
Scopus	97	96		21	214
ACM	8	11		0	19
Elsevier	13	0		0	13
IEEE	23	64		0	87
Springer	17	33		0	50
	198	204		27	**429**
**Selection by Document Title (ST2)**					
	**Journal/Magazine**	**Conferences/Book Chapters**		**Reviews**	**Total**
ISI	8	0		0	8
Scopus	60	104		10	174
ACM	12	21		0	33
Elsevier	16	0		0	16
IEEE	19	126		0	145
Springer	15	50		0	65
	130	301		10	**441**
**Duplicate Detection and Removal**					
**ST1**	**Total by Type**	**Duplicated**	**Final Set**	J1: Non-duplicated results from journals, magazines and reviews. C1: Non-duplicated results from conferences or book chapters.	
Journal/Magazine	198	19	179		
Conferences/Book chapters	204	0	204		
Reviews	27	3	24		
	429	22	**407**		
**ST2**	**Total by Type**	**Duplicated**	**Final Set**	J2: Non-duplicated results from journals, magazines and reviews. C2: Non-duplicated results from conferences or book chapters.	
Journal/Magazine	130	5	125		
Conferences/Book chapters	301	20	281		
Reviews	10	0	10		
	441	25	**416**		

**Table 3:** Merging results from ST1 and ST2.

**Merged Results from all Search Strings**				
	**ST1**	**ST2**	**Total**	
Journal/Magazine/Reviews	203	135	338	
Conferences/Book chapters	204	281	485	
	407	416	**823**	
**Duplicate Detection and Removal**				
	**Total by Type**	**Duplicated**	**Final Set**	$J=J_{1}\cup J_{2}$ $C=C_{1}\cup C_{2}$
Journal/Magazine/Reviews	338	92	246	
Conferences/Book chapters	485	157	328	
	823	249	**574**	

**Table 4:** Primary studies selection.

**Set**	**Total**	**F1**	**F2**	F1: Filter by abstract and keywords. F2: Skimming. Remaining documents are final sets PJ and PC.
J	246	68	**36**	
C	328	57	**39**	

**Table 5:** Joy’s six Webs taxonomy.

Category	Description
Near Web	It refers to the version of the Web that is closer to us, which is accessed using a computer by means of interfaces like keyboards and mice. It is defined by information and provides a notion of mobility through wireless networks.
Here Web	The version of the Web that can be accessed anytime and from any place using a device that is always with a person, becoming part of his or her identity.
Far Web	This Web refers to accessing contents through broadband networks. Such contents usually infer an innovation in entertainment.
Weird Web	The Web that is accessed with natural user interfaces. Its style of use defines it as the most pervasive of the first four Webs.
B2B	In this version of the Web, business computers talk to each other about business processes. It was initially identified as “e-commerce Web”.
D2D	The device-to-device Web refers to devices communicating to share information to manage, control and monitor processes. It was initially identified as “pervasive Web”.
